# The Modified Extended Fleur-De-Lis Latissimus Dorsi Flap for Various Complex Multi-directional Large Soft and Bone Tissue Reconstruction

**DOI:** 10.7759/cureus.6974

**Published:** 2020-02-12

**Authors:** Pedro Ciudad, Oscar J Manrique, Samyd S Bustos, Georgios Pafitanis, Antonio J Forte, Maria T Huayllani, Daniel Boczar, Maria Vargas, Silvia Escalante, Luis Parra Pont, Atenas Bustamante, Hung-Chi Chen

**Affiliations:** 1 Plastic, Reconstructive and Burn Surgery, Arzobispo Loayza National Hospital, Lima, PER; 2 Plastic Surgery, Mayo Clinic, Rochester, USA; 3 Plastic and Reconstructive Surgery, China Medical University Hospital, Taichung, TWN; 4 Plastic Surgery, Mayo Clinic Florida - Robert D. and Patricia E. Kern Center for the Science of Health Care Delivery, Jacksonville, USA

**Keywords:** latissimus dorsi flap, extended fleur-de-lis latissimus dorsi flap, multi-directional tissue defect, complex reconstruction, complications

## Abstract

Introduction

Latissimus Dorsi (LD) myocutaneous flap is a workhorse flap for various large reconstructions. Variants described to increase its volume are referred to as extended LD flaps. The extended fleur-de-lis LD is one of these variants. We report the clinical outcomes using a modified extended fleur-de-lis LD flap for complex multi-directional soft and bone tissue defects.

Methods

Between 2010 and 2017, 29 patients underwent the modified extended fleur-de-lis LD flaps, whose sizes were between 120 cm^2 ^and 442 cm^2^. The mean age was 47.55 ± 9.07 years. Locations of the defects included head and neck (nine cases), upper extremity (six cases), lower extremity (nine cases) and chest wall regions (five cases).

Results

Of the 29 extended fleur-de-lis LD flaps, 11 were pedicled and 18 were free flaps. A total of 10 flaps were osteomyocutaneous and 19 were myocutaneous. The mean vertical size of the harvested skin paddle was 30.72 ± 4.57 cm (range: 20-38), and the mean horizontal size of the skin paddle was 8.69 ± 0.80 (range: 7-10) cm, with the mean maximum horizontal extensions of the flaps being 16.03 ± 1.18 (range: 14-18) cm. The flap survival rates were 100%. One flap had distal partial loss (less than 5%). Donor site complications included seroma (1) and numbness (1), both of which were managed conservatively. The average follow-up time was 23.97 ± 7.19 months.

Conclusion

The presented modified extended fleur-de-lis LD myocutaneous/osteomyocutaneous flap for reconstruction of multi-directional complex soft tissue and bone defects is a good and reliable option, with low donor site morbidity.

## Introduction

The surgical management of complex wound defects remains a challenge for reconstructive microsurgeons. Extensive soft tissue defect reconstruction can be achieved by utilizing several techniques, including those that involve a large single flap, multiple flaps or even combined linking flaps [1-2]. Although these techniques can potentially provide superior reconstructive outcomes, complex and large wound defect reconstruction requires demanding microsurgical techniques and presents concerns for the morbidity of donor sites and associated complications [3]. One-stage single flap procedures offer safer and lower risk options [4]. However, donor site options for large one-stage single flap reconstruction are limited by the three-dimensional characteristics and composition of tissue required in extensive and complex wound defects [5].

The latissimus dorsi (LD) myocutaneous flap, since its first description by Tanzini in 1906 [6], has been used as an option for several reconstruction modalities. It is a pliable and robust flap that can cover large tissue defects [7]. Its two-dimensional, flexible characteristics and vascularity enable reconstruction of broad defects and offer a moderately large single flap option. For bulky defects, a transverse rectus abdominis myocutaneous (TRAM) flap could be considered; however, it may cause abdominal wall hernias in the donor site [8-9]. Various modifications to increase the volume of the flap have been proposed by many authors [8-9]. These LD flap variants have been designated as extended LD myocutaneous flaps and were first described for breast reconstruction. However, they have also been used for reconstructions of large, complex soft tissue defects in other parts of the body [10]. The extended fleur-de-lis LD flap is one of these variants and was first introduced by McGraw and Papp in 1991 for breast reconstruction, but later it was applied in other reconstructions as well [9, 11-12].

This study aimed to report our experience with the extended fleur-de-lis LD myocutaneous/osteomyocutaneous flap for different large and complex multi-directional reconstructions.

## Materials and methods

Between 2010 and 2017, 29 extended fleur-de-lis LD flaps were used for the reconstruction of various complex tissue defects. The inclusion criteria in this study were patients who had large soft tissue defects on the head and neck, extremities, or thorax regions with suitable donor sites. Patients who had a previous LD flap, scar or trauma at the donor site were excluded from the study. The age of the participating patients ranged between 32 and 69 (mean age: 47.55 ± 9.07 years). Defects to be reconstructed were on the head and neck (9), upper extremity (6), lower extremity (9) and thorax (5). The mean size of the defects was 266.34 ± 85.03 cm^2 ^(120 cm^2 ^to 442 cm^2^). Table 1 lists the various causes of the defects, which include 11 cases of trauma, nine cases of tumour or metastasis excision, three cases of arteriovenous malformation excision, three cases of neck contracture, two cases of chronic infection and one case of osteoradionecrosis.

**Table 1:** Defects and data of patients who received extended fleur-de-lis latissimus dorsi myocutaneous flap reconstruction Abbreviations: SD: standard deviation; Min: minimum; Max: maximum; AVM: arteriovenous malformation

Characteristics	Categories	No. of patients
Gender		
	Male	23
	Female	6
Age (years)		
	Min	32
	Max	69
	Mean	47.55
	SD	9.07
Defect Size of defects (cm^2^)		
	Min	120
	Max	442
	Mean	266.35
	SD	85.03
Location of defects		
	Head and Neck	9
	Upper Extremity	6
	Lower Extremity	9
	Thorax	5
Cause of defects		
	Trauma	11
	Tumor or metastasis excision	9
	AVM excision	3
	Neck contracture	3
	Chronic Infection	2
	Osteoradionecrosis	1

Surgical technique

Design and Markings

**Figure 1:**
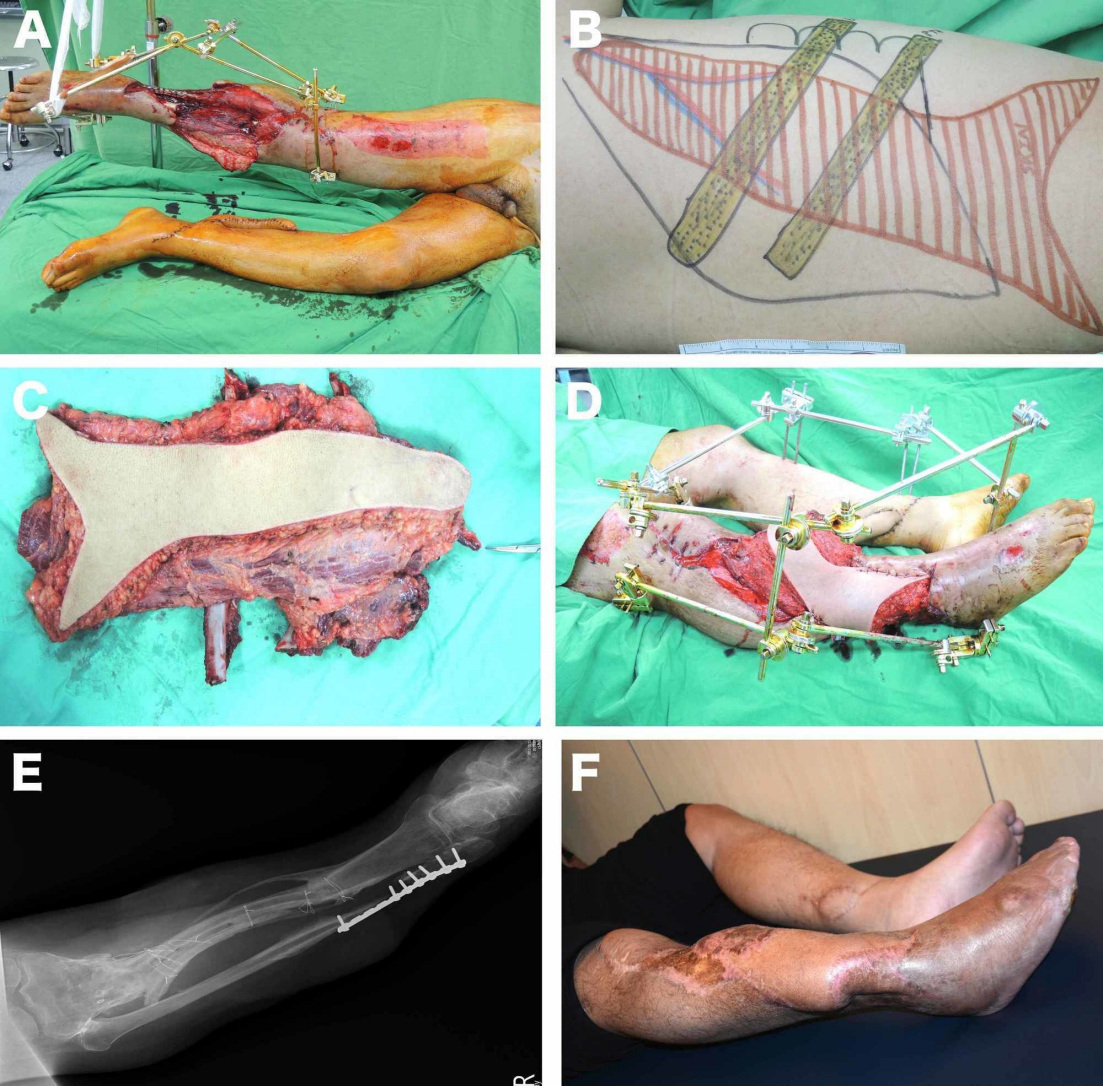
Lower extremity reconstruction with free extended fleur-de-lis LD osteomyocutaneous flap (A) A 26 x 17 cm tissue defect on the right leg due to injury sustained in a motor vehicle accident; (B) The design of extended fleur-de-lis LD osteomyocutaneous flap involving 7th and 9th ribs (the vertical dimension of the flap is 35 cm and the horizontal dimension of the extension is 15 cm); (C) Harvested flap; (D) Flap inset; (E) X-ray, at one-year postoperative, showing the bony integration; (F) The appearance of the leg at the three-year postoperative evaluation.

**Figure 2:**
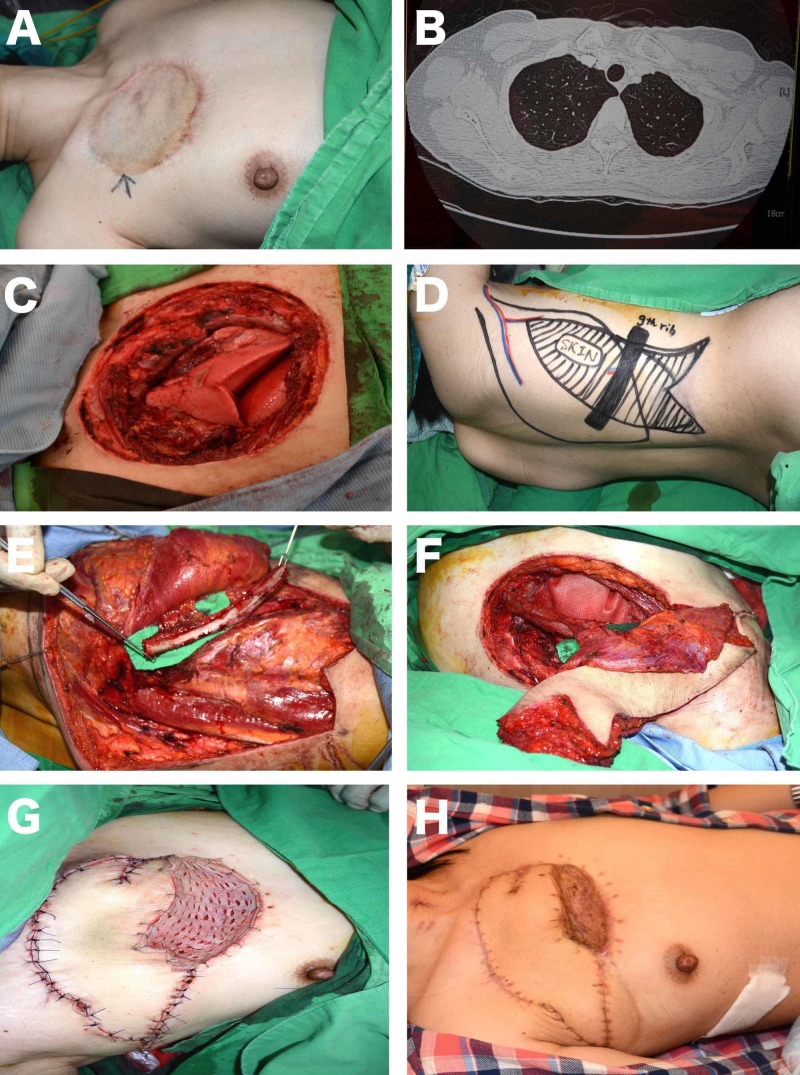
Chest wall reconstruction with pedicled extended fleur-de-lis LD osteomyocutaneous flap (A) Skin grafted area due to previous excision of lipoangiosarcoma on the left anterior chest wall of a 55-year-old female patient; (B) Axial view of the thorax on computerized tomography showing the recurrence three years after the first excision; (C) Wide excision, involving first, second and third ribs, exposing the lung (defect size is 18 x 14 cm); (D) The design of the pedicled extended fleur-de-lis LD osteomyocutaneous flap. The distal medial and lateral triangular extensions are just above the posterior-superior iliac crest; (E) The ninth rib was included in the flap; (F) Harvested flap; (G) The distal skin extensions were positioned parallel to the vertical axis of the flap in order to lengthen the flap, and the muscular part of the flap was used to cover the dead space; (H) The patient, without recurrence, after two years.

With the patient in prone or lateral decubitus position, the skin island was marked in a fleur-de-lis design in the area overlying the LD muscle. This marked skin island consisted of a long, extended vertical skin island along the reliable LD perfusion region and two triangular medial and lateral extensions (the horizontal skin island part) above posterior superior iliac crest (PSIC). The triangular extensions offered a distal inferior flap skin and a volume below the conventional 8 cm from PSIC (Figures 1-2). This modified design allowed for maximization of the skin paddle in a multi-directional manner, and the pinch test in two dimensions was utilized to confirm the feasibility for primary donor site closure. ​​​​​​

Flap Dissection

The anterior border of the vertical skin island was incised away from the skin island to identify the anterior border of the LD muscle. Initial dissection of the superficial surface of the muscle along the posterior axillary line, with a window bridging the fascia overlying the muscle, allowed the space between the LD muscle and the thoracolumbar fascia to be entered. Careful dissection and ligation of inter-costal perforators posteriorly and dis-insertion of the muscle distally from the iliac crest were performed, and the insertions were made in the midline. The inferior triangular incisions with a full thickness dissection deep to the thoracolumbar fascia ensured the preservation of fascial vascular supply to the distal part of the flap. Dissection distal to proximal, with careful ligation of thoracolumbar perforators, was vital to avoid complications. The flap was elevated to reach the superior fibers of the muscle and the upper tendinous origin, which could be located under the inferior trapezius muscle fibers. These superior medial fibers of origin were then divided. The LD muscle was taken with the underlying paraspinous muscle fascia. If the flap would include a rib segment, a patent musculoperiosteal perforator vessel was identified and isolated to transfer the rib based on the periosteal blood supply. Then, the desired segment of the rib was osteotomized laterally and medially, leaving a 3-4 cm of rib segment on each side. Dissection was carefully continued anteriorly to separate the LD from the serratus muscle posterior-lateral, where it is fused. The entire flap was then elevated towards the axilla in an oblique fashion. The superior muscle was divided from its insertion. Axillary dissection with ligation of the small branches of the pedicle was important to achieve adequate length.

Flap Inset

Flap inset could be done according to the requirements of the tissue defects. To cover vital structures, the vertical skin paddle and the horizontal extensions could be positioned in multi-directional ways. Besides this, the length of the flap could be extended by positioning the horizontal skin paddles vertically, parallel to the vertical skin paddle, or the bulk of the flap could be augmented by folding the distal horizontal parts on the vertical skin paddle. During the inset of the flap, it was critically important to avoid tightness, especially at the distal part of the flap. When a primary closure could not be accomplished, a partial skin graft was harvested from the leg in order to avoid tension of the recipient area. As the distal extension had only fascia-cutaneous blood supply, vascularization of this part could easily be compromised due to tight closure. 

Donor Site Closure and Postoperative Period

Donor site closure was done primarily in an inverted Y fashion. Two active Jackson-Pratt drains were inserted and kept for at least two weeks at the donor site. The patients remained in the intensive care unit for approximately seven days, and were able to be discharged from the hospital two weeks after the operation. The follow-up period changed according to the patients. In the first month after the operation, patients typically underwent check-ups in the outpatient clinic once a week, and after the first month, once a month within the first postoperative year.

## Results

**Figure 3:**
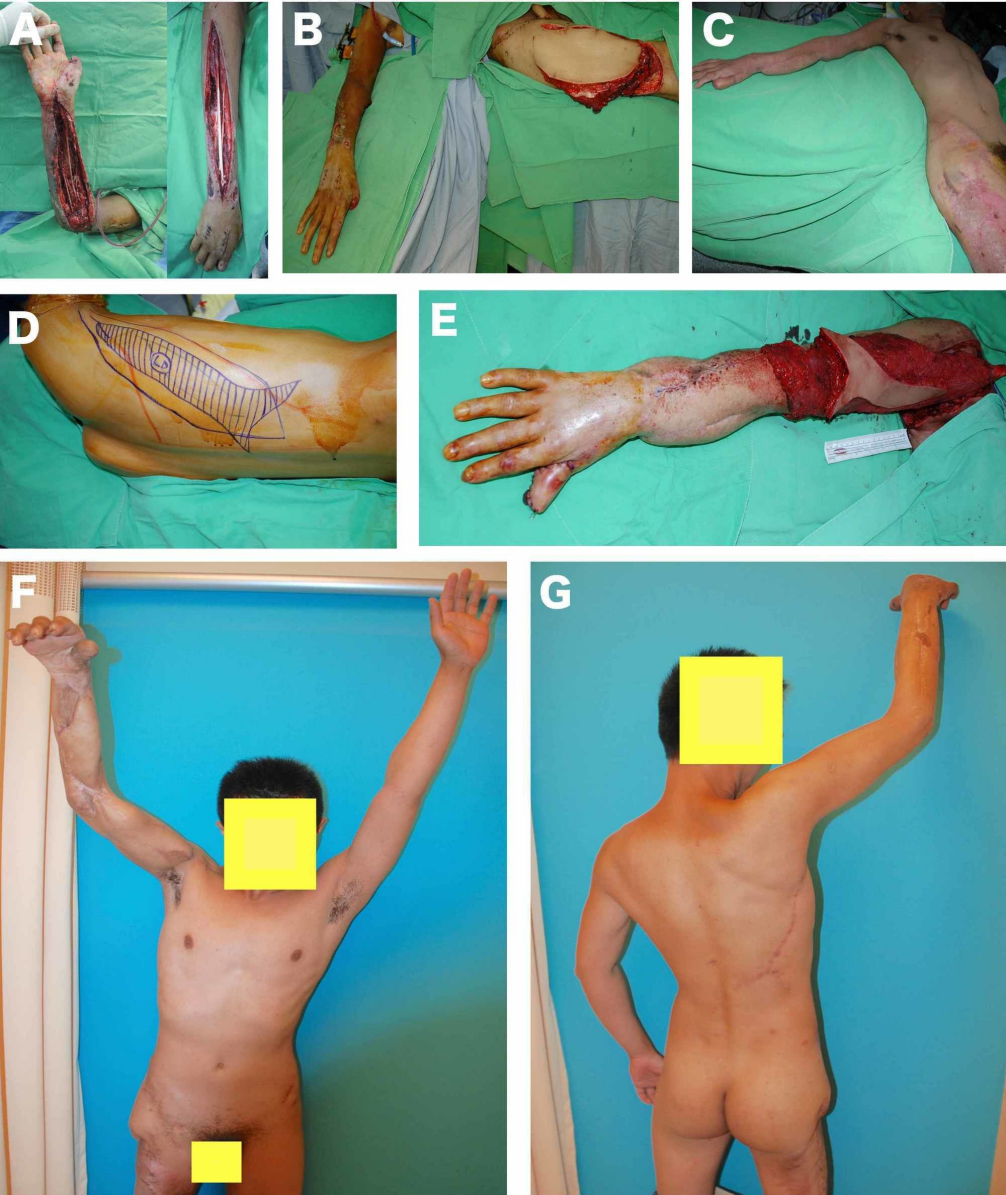
Extended fleur-de-lis LD musculocutaneous flap for functional muscle transfer (A) Compartment syndrome due to severe crush injury of the right forearm and arm (B) The exposed flexor tendons of the hand on the arm covered with free anterolateral thigh flap at the first stage; (C) The second stage of the operation, performed three months after the first operation to reconstruct the elbow flexion; (D) The planning of the extended fleur-de-lis LD musculocutaneous (vertical size is 35 cm, horizontal size of the distal extensions is 15 cm); (E) The inset of the pedicled LD flap; (F) Frontal view of the patient moving his right arm and forearm at three-years postoperative; (G) Dorsal view of the patient moving his right arm and forearm at the three-year postoperative evaluation.

**Figure 4:**
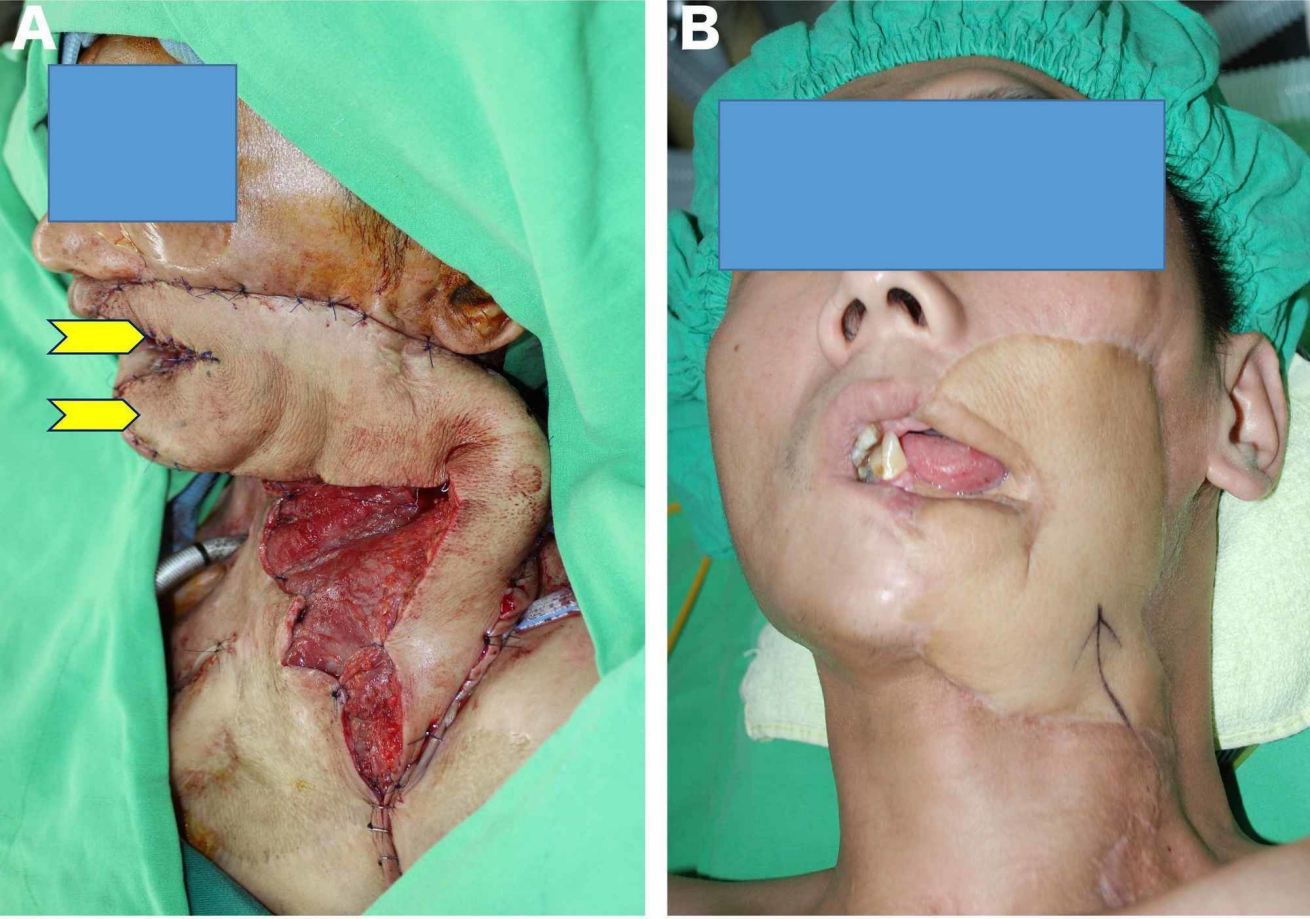
Extended fleur-de-lis LD musculocutaneous free flap for multi-directional defect (A) Intra-operative picture showing (yellow arrows) inset of the medial and lateral triangular distal extensions of the flap separately to replace tissue defects on the upper lip and lower lip and chin; (B) Postoperative follow-up at eight months.

**Figure 5:**
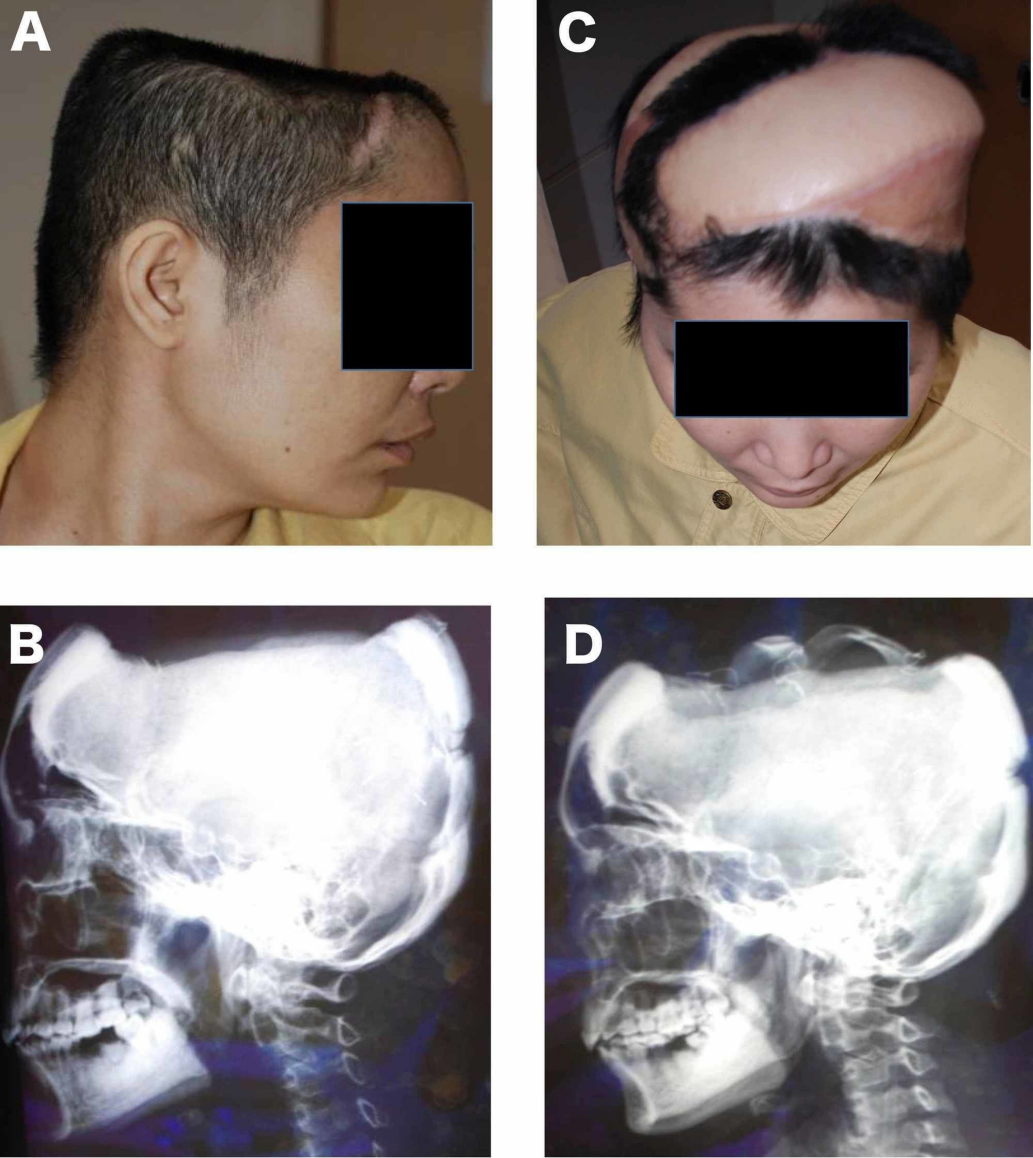
Scalp and calvarium reconstruction with extended fleur-de-lis LD musculocutaneous free flap (A) Preoperative view of the patient; (B) Preoperative X-ray; (C) Postoperative view; (D) Postoperative X-ray showing the calvarium reconstruction with two ribs.

There were eleven defects reconstructed by pedicle extended fleur-de-lis latissimus dorsi flap, but for 18 patients, the extended fleur-de-lis latissimus dorsi flap was used as free flaps. For these cases, the recipient vessels were the transverse cervical vessels (TCV) (seven cases), superficial temporal vessels (STV) (two cases), contralateral posterior tibial vessels (PTV) (four cases), medial sural vessels (MSV) (three cases) and medial plantar vessels (MPV) (two cases). The mean vertical size of the harvested skin paddle was 30.72 ± 4.57 cm (range: 20 cm to 38 cm), the mean horizontal size of the skin paddle was 8.69 ± 0.80 cm (range: 7 cm to 10 cm), and the mean maximum size of the horizontal extensions of the flaps was 16.03 ± 1.18cm (range, 14 cm to 18 cm). Flap survival rates were 100% in 28 flaps and 95% in one flap with no exploration. There were a few minor complications at the donor sites, including one seroma and one patient with permanent numbness. Seroma was successfully managed with aspiration and compression. Only minor complications were seen at the recipient site, including one case of hematoma, two cases of partial skin graft loss and one case of wound dehiscence. Debridement and seconder suturing were performed under local anesthesia for wound dehiscence. The average follow-up was 23.97 ± 7.19 months (range: 14 to 36 months). After the healing period, none of the patients had complaints about pain or weakness on their shoulders or about the appearance of the donor areas, although range of motion and strength testing were not completed. The donor site contour and scars were acceptable for the patients. The results are summarized in detail in Table 2 and shown in Figures 1-5.

**Table 2:** Extended fleur-de-lis latissimus dorsi myocutaneous flap characteristics Abbreviations: FMT: Functional muscle transfer; MC: Myocutaneous; OMC: Osteomyocutaneous; TCV: Transverse cervical vessels; STV: Superficial temporal vessels; PTV: Posterior tibial vessels; MSV: Medial sural vessels; MPV: Medial plantar vessels

Recipient area		Procedure	Type of flap	Pedicled/Free	Recipient vessels	Skin paddle vertical size (cm)	Skin paddle horizontal size (cm)	Horizontal extension max size (cm)	Flap survival (%)	Complication		Follow-up (months)	Outcomes
										Donor	Recipient		
Head and Neck													
	1	Soft tissue coverage	MC	Free	TCV	25	9	15	100	None	None	15	Stable Soft Tissue Cover
	2	Soft tissue coverage	MC	Free	TCV	28	8	14	95	None	Wound dehiscence	21	Stable Soft Tissue Cover
	3	Soft tissue coverage	MC	Free	TCV	25	9	15	100	None	None	16	Stable Soft Tissue Cover
	4	Tissue augmentation / contracture release	MC	Free	TCV	25	9	17	100	None	None	18	Supple Neck
	5	Contracture release	MC	Free	TCV	28	9	17	100	None	None	19	Supple Neck
	6	Cranium / scalp reconstruction	OMC	Free	STV	25	8	16	100	None	Distal Epidermolysis	36	Stable Soft Tissue Cover and Bone Stability
	7	Contracture release	MC	Free	TCV	20	9	16	100	None	None	14	Supple Neck
	8	Cranium / scalp reconstruction	OMC	Free	STV	25	10	17	100	None	None	18	Stable Soft Tissue Cover and Bone Stability
	9	Mandible reconstruction	OMC	Free	TCV	25	8	16	100	None	None	36	Stable Mandibular Continuity
Upper extremity													
	1	FMT	MC	Pedicled		35	7	15	100	None	None	36	Mobility
	2	FMT	MC	Pedicled		32	8	14	100	None	Hematoma	32	Mobility
	3	FMT	MC	Pedicled		38	7	15	100	None	None	18	Mobility
	4	FMT	MC	Pedicled		32	8	16	100	None	None	21	Mobility
	5	FMT	MC	Pedicled		30	8	15	100	None	None	24	Mobility
	6	FMT	MC	Pedicled		32	7	14	100	None	None	26	Mobility
Lower extremity													
	1	Bone /soft tissue coverage	OMC	Free	Contralateral PTV	35	9	17	100	Seroma	none	36	Stable Soft Tissue Cover and Bone Stability
	2	Soft tissue coverage	MC	Free	MSV	33	9	18	100	None	None	24	Stable Soft Tissue Cover
	3	Bone /soft tissue coverage	OMC	Free	Contralateral PTV	38	9	16	100	None	None	25	Stable Soft Tissue Cover and Bone Stability
	4	Bone / soft tissue coverage	OMC	Free	MSV	31	9	17	100	None	None	18	Stable Soft Tissue Cover and Bone Stability
	5	Soft tissue coverage	MC	Free	MSV	32	9	15	100	None	None	26	Stable Soft Tissue Cover
	6	Bone / soft tissue coverage	OMC	Free	Contralateral PTV	30	9	17	100	None	Partial skin graft loss	28	Stable Soft Tissue Cover and Bone Stability
	7	Soft tissue coverage	MC	Free	MPV	33	9	18	100	None	None	30	Stable Soft Tissue Cover
	8	Bone / soft tissue coverage	OMC	Free	Contralateral PTV	35	9	16	100	None	Partial distal necrosis	15	Stable Soft Tissue Cover and Bone Stability
	9	Soft tissue coverage	MC	Free	MPV	31	9	15	100	Numbness	None	20	Stable Soft Tissue Cover
Thorax													
	1	Bone / soft tissue coverage	OMC	Pedicled		35	9	16	100	None	None	24	Stable Chest Wall
	2	Soft tissue coverage	MC	Pedicled		32	9	16	100	None	None	34	Stable Chest Wall
	3	Bone / soft tissue coverage	OMC	Pedicled		30	9	17	100	None	None	23	Stable Chest Wall
	4	Soft tissue coverage	MC	Pedicled		33	10	18	100	None	None	28	Stable Chest Wall
	5	Soft tissue coverage	MC	Pedicled		38	10	17	100	None	Partial skin graft loss	14	Stable Chest Wall

Case Reports

Case 1

A 37-year-old male was referred to our clinic after sustaining a severe crush injury to the right leg due to a traffic accident. The first intervention was performed in another hospital, where the open fracture of the tibia had been fixed by an external fixator, but there was a segmental loss of the tibia and a large soft tissue defect (26 x 17 cm) (Figure 1A). The free extended fleur-de-lis LD osteomyocutaneous flap, which included the seventh and ninth ribs, was chosen to cover the large soft tissue defect as well as to replace the segmental tibial loss. The flap’s vertical skin island was 35 cm, the horizontal skin island was 9 cm, and the maximum size of the horizontal extensions was 17 cm (Figures 1B-1D). The distal extensions of the flap were used to cover the exposed distal tibia and fibula. The perioperative period was without incident. During the follow-up period, seroma collection on the donor site was detected, but it was successfully managed through conservative measures in the clinic, without the need for another intervention. At 12 months of follow-up, the patient was able to walk (Figures 1E-1F).

Case 2

A 55-year-old female patient with a lipoangiosarcoma in her right chest wall presented to our clinic. Three years prior to her presentation at our clinic, a mass diagnosed as lipoangiosarcoma had been excised from her right anterior chest wall and reconstructed via a split-thickness skin graft (Figure 2A) There was a recurrence of the tumor invading the first, second, and third rib. In addition, the pleura was also invaded (Figure 2B). A large excision, which involved the ribs and pleura, was performed, after which the lung was exposed and the size of the defect was determined to be 18 x 14 cm (Figure 2C). In order to cover this defect and to reestablish the chest wall stability, a pedicled extended fleur-de-lis LD osteomyocutaneous flap, which included the ninth rib, was used (Figures 2D-2F). The flap’s vertical skin island was 35 cm, the horizontal skin island was 9 cm, and the maximum size of the horizontal extensions was 16 cm. The distal extensions were used to elongate the vertical length of the flap, and a split-thickness skin graft was used to cover the muscular part of the flap, which was filling the dead space (Figure 2G). The healing period was uneventful, and to date, the patient has reached the 24th month of the follow-up period without any sign of recurrence (Figure 2H).

Case 3

A 36-year-old male patient who sustained a severe crush injury to his right upper extremity was referred to our clinic after first being treated at another hospital. A serious case of compartment syndrome was detected on the forearm and upper arm of the patient. The flexor tendons of the hand were exposed and necrotic on the volar forearm, and there was a soft tissue defect on the dorsal side (Figure 3A). The reconstruction for this complex trauma case was done in two stages. At the first stage, the flexor tendons on the volar arm were covered with the free chimeric anterolateral thigh flap and vastus lateralis muscle flap, while the dorsal side was covered with a split-thickness skin graft (Figure 3B). The second stage was performed three months later when the wound healed completely. A functional muscle transfer was needed to restore the flexion of the elbow. At the second stage, the scar tissue on the volar and dorsal sides of the arm had to be released first and it resulted in a soft tissue defect of 20 x 12 cm. A pedicled extended fleur-de-lis LD myocutaneous flap was chosen to restore this defect, as well as to restore the flexion function of the elbow (Figure 3C). The vertical size of the flap was 35 cm, the horizontal size of the defect was 7 cm and the maximum distal skin paddle size was 15 cm. The LD muscle was used for restoration of the flexion function, and skin islands were applied to cover the soft tissue defect (Figures 3D-3E). The healing and follow-up (36 months) period was uneventful, and the patient was able to extend his forearm (Figures 3F-3G).

## Discussion

The LD myocutaneous flap has been used as an option for several types of reconstructions, especially as a workhorse pedicle flap for breast reconstructions [6]. Many authors have reported on its use as a pedicle or free flap in other large soft tissue reconstructions, such as head and neck reconstructions, lower and upper extremity reconstructions and trunk reconstructions [7, 13-15]. The LD myocutaneous flap offers a constant and versatile vascular anatomy, and a discrete donor site with low morbidity. Another advantage of this flap is its proximity to the breast, chest, back, and even head and neck area, so it can be transposed for the reconstructions of these areas as a pedicle flap without the need of a microvascular anastomosis [6, 13]. Their major drawback is their inability to repair deep defects, as they require an extensive volume of tissue replacement because the relatively large muscle surface area does not have enough volume for bulky reconstructions of deep defects [8]. Several modifications of the traditionally described LD myocutaneous flap have been described and utilized to overcome this drawback. The term “extended” LD flap was coined to describe larger tissue transfers. At first, the new term applied to a tissue transfer involving breast reconstruction, but was later expanded to include other reconstructions as well [10, 16-19]. In this study, we demonstrated the application of a distal modification, wherein, using a multi-directional design, the size and volume of the conventional LD flap was able to be increased. In this case series, the distal modification was used for twenty-nine large, complex and multi-directional soft tissue reconstructions on the head and neck, upper extremity, lower extremity and thorax regions. A fleur-de-lis flap design has a secondary horizontal skin paddle to the distal end of the main vertical skin paddle, with medial and lateral extensions. The aim of this design was to increase the surface area of the skin paddle as well as to enlarge the volume of the flap in a versatile, multi-directional manner for complex reconstructions. By keeping the vertical skin paddle for as long as possible and extending the distal horizontal skin paddles medial and lateral over the thoracolumbar fascia, this design offers distal flap bulk. In this series, 11 LD myocutaneous/osteomyocutaneous flaps were used as pedicle flaps, six for upper extremity reconstructions and five for thorax reconstructions. 

The reconstruction of large, complex soft tissue defects can be very challenging, with many alternatives having been attempted to repair these defects. One of these options is covering the wound with a large single flap. There are a few suitable donor sites for large flaps that include the thigh region for anterolateral thigh (ALT) flap and the lower abdominal area for rectus abdominis muscle myocutaneous flaps or deep inferior epigastric perforator (DIEP) flaps [20]. The literature presents some cases of very large flaps from these donor areas (e.g. 40 x 20 cm^2^ ALT flap and 50 x 17 cm^2 ^DIEP flap) [21-22]. The major drawback of using these very large flaps is donor site morbidity. Even if no other complication occurs, the cosmetic appearance of donor sites will be poor on account of needing to cover them with skin grafts [23]. One of the most significant advantages of using our technique is the minimal morbidity created by the donor area. In our series, we managed to close the donor sites of even very large flaps (440 cm^2^), generally without any major complications. Previous series reported that skin grafting is required for donor sites of extended latissimus variants [10]. However, with this design presented here, the tension in the donor area can be spread over two different axes, making it always possible to close the donor area in the form of an inverted Y fashion, and the use of skin graft is no longer necessary. By using the extended fleur-de-lis LD flap, aesthetically acceptable results were obtained in the donor areas of all patients.

Other alternatives suggested in the literature for closing large tissue defects include the use of multiple conventional flaps or the use of a combination of linked and chimeric perforator flaps [2-4]. Multiple flaps provide a great advantage, particularly in closing complex multi-directional defects. Very successful three-dimensional reconstructions can be made through the combination of linked and chimeric perforator flaps [3, 4]. It is not possible to cover multi-directional defects with one large uni-directional flap. Therefore, positioning multiple flaps in the proper positions, with flexibility, makes it possible to cover these defects.

While good results have been reported for repairs of large defects and for repair of multiple defects using multiple flaps, this method has significant disadvantages [24]; the use of more than one flap involves multiple donor site morbidities as well as extra surgical risks associated with each flap. The harvesting of these flaps is technically very demanding and their vascularity is dependent on angiosomes [25]. In multi-directional soft tissue repair, the modified extended fleur-de-lis LD myocutaneous flap succeeded in overcoming all the problems that characterize the use of multiple flaps. A single flap was used for reconstruction in each case, with only one flap donor area needed (i.e. there was no need of an additional flap donor area). Repair of multiple dimensional tissue defects was overwhelmed via the recommended versatile flap design. By positioning the vertical and horizontal parts of the flap in different figures, the complex defects that had different axes were able to be easily repaired without the need for an additional flap (Figure 4). Moreover, positioning the vertical and horizontal parts in different directions not only made it possible to repair multi-directional defects. The harvest was also relatively easy, as the proposed modification did not require a more complicated method than that of a conventional LD ​​myocutaneous flap harvest. In our series, there were no cases requiring combined or chimeric perforator flaps based in thoracodorsal vessels. One other advantage that the modified extended LD fleur-de-lis myocutaneous flap has over other large flaps, which do not contain muscle, is its ability to be used for functional muscle restoration. In our series, six flaps were effectively transferred as functional muscles to repair both bulky soft tissue defects as well as muscle functions (Figure 3).

Reconstruction of large soft tissue defects that also require replacement of the bone tissue is also a challenging problem. The latissimus muscle flap and the latissimus dorsi musculocutaneous composite flap that include the ribs have been previously described and used for various bone tissue replacements [26]. The most significant advantage of the latissimus dorsi osteomyocutaneous flap over other flaps commonly used for bone reconstruction, like the fibula osseocutaneous flap, radial forearm osseocutaneous flap, and iliac crest osteomyocutaneous flap, is the large soft tissue volume that it has [27]. Hence, it is very suitable for the repair of large soft tissue defects that require bone replacement. In this series, the new modification of the extended fleur-de-lis skin paddle design was successfully used as osteomyocutaneous composite flaps for three head and neck, four lower extremity and two chest wall reconstructions that required bone replacement and extensive soft tissue coverage (Figures 1-2, 5). In addition, the current series is one of the largest describing the modified extended fleur-de-lis LD osteomyocutaneous flap.

One of the biggest concerns about the use of the extended fleur-de-lis LD myocutaneous flap is related to the vascularity of the skin island at the distal end. To resolve this concern when using the pedicle version, lumbar perforator flaps can be preserved, as we reported earlier [12]. The contribution of the fasciocutaneous plexus to the vascularity of the skin island of flaps has been clearly demonstrated [28]. When using the extended fleur-de-lis LD myocutaneous as a free flap, careful attention was paid to the preservation of the connection between the skin island located at the distal extension and the thoracolumbar fascia. Although there had been some signs of venous congestion in the early stage after the elevation of flaps, there was no skin necrosis, even in the flaps where the skin islands were extended to the lumbar and posterior iliac crest area. Another important point that needs to be considered to prevent vascular compromise at distal area, is to avoid tight closure during the inset of the flap. In our series, there were one case with epidermolysis and one partial necrosis at the distal ends of the flap and one case of wound dehiscence. Fortunately, these were managed by simple debridement and secondary suturing, without the need for a more extensive intervention.

Seroma in the donor area is one of the disadvantages of the LD myocutaneous flap [29]. Quilting sutures with fibrin glue application and triamcinolone injections are recommended for the prevention and treatment of seroma in the donor areas of extended latissimus LD myocutaneous flap [30]. In our series, we had only one seroma in the donor areas, and it was treated by simple aspiration, without the need for further interventions. The low seroma frequency in our series could be attributed to the use of quilting sutures during donor site closures in addition to the tight tension closure due to the large skin islands harvested.

## Conclusions

The new modified extended fleur-de-lis LD myocutaneous/osteomyocutaneous flap has been successfully used in the reconstruction of complex soft tissue defects located on various parts of the body. With this new modification, major issues in the reconstruction of complex soft tissue defects are likely to be resolved. First, it is possible to repair large defects with a single flap by extending the distal horizontal parts vertically to obtain a long flap. Second, defects requiring a greater volume can be reconstructed by folding the distal horizontal part on itself. Lastly, it is possible to reconstruct multi-directional defects with a single flap by positioning vertical and horizontal parts in different combinations. The extended fleur-de-lis LD myocutaneous flap has advantages over multiple flaps or the combination of linked and chimeric flaps in terms of having less morbidity.
